# Supplementary figures and images for: State-level variation of initial COVID-19 dynamics in the United States
Source: PLoS One. 2020 Oct 13;15(10):e0240648. doi: 10.1371/journal.pone.0240648 (PMC7553297; doi:10.1371/journal.pone.0240648)

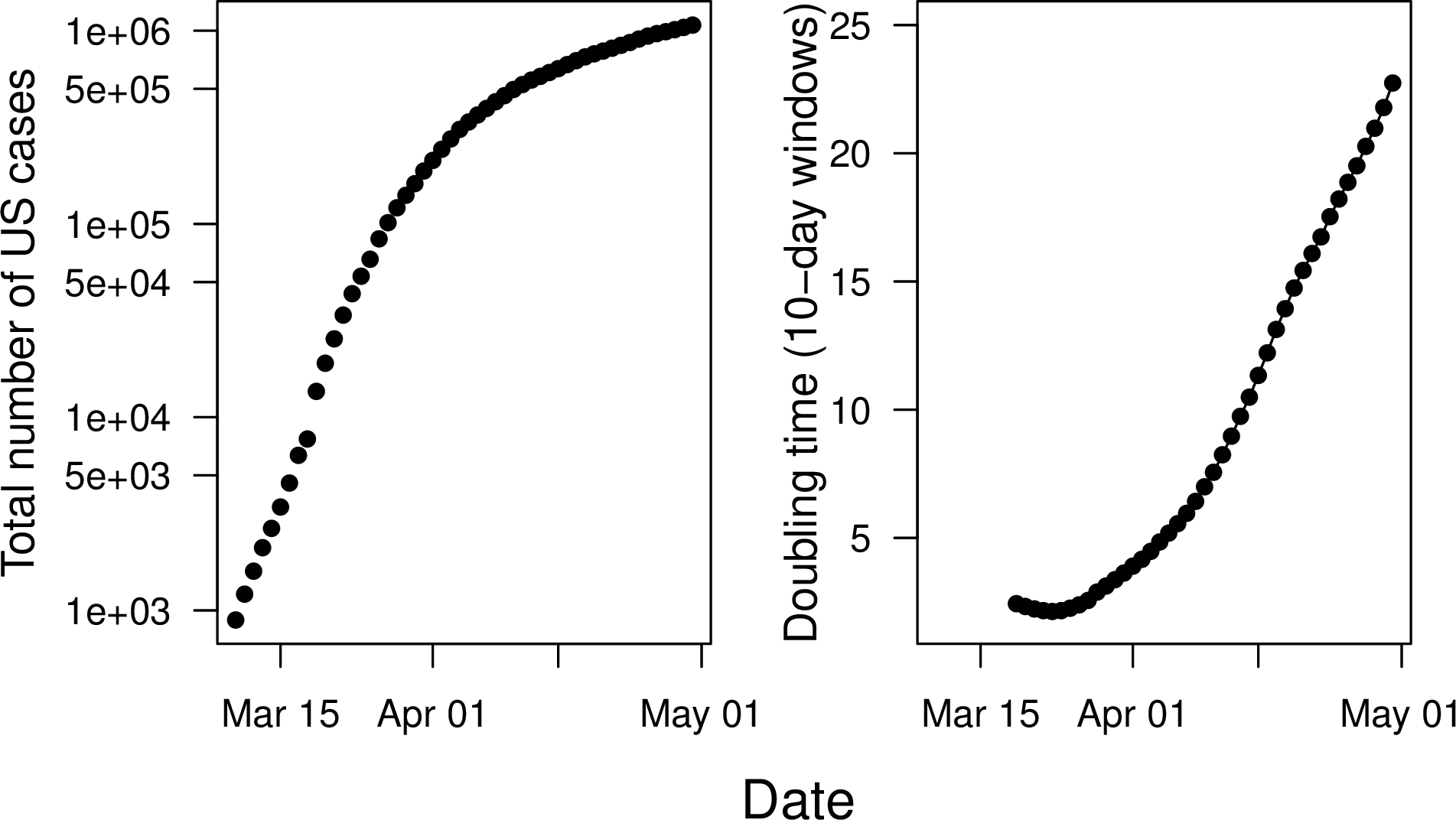

Supplement: S1 Fig — (TIF) [file pone.0240648.s001.tif]

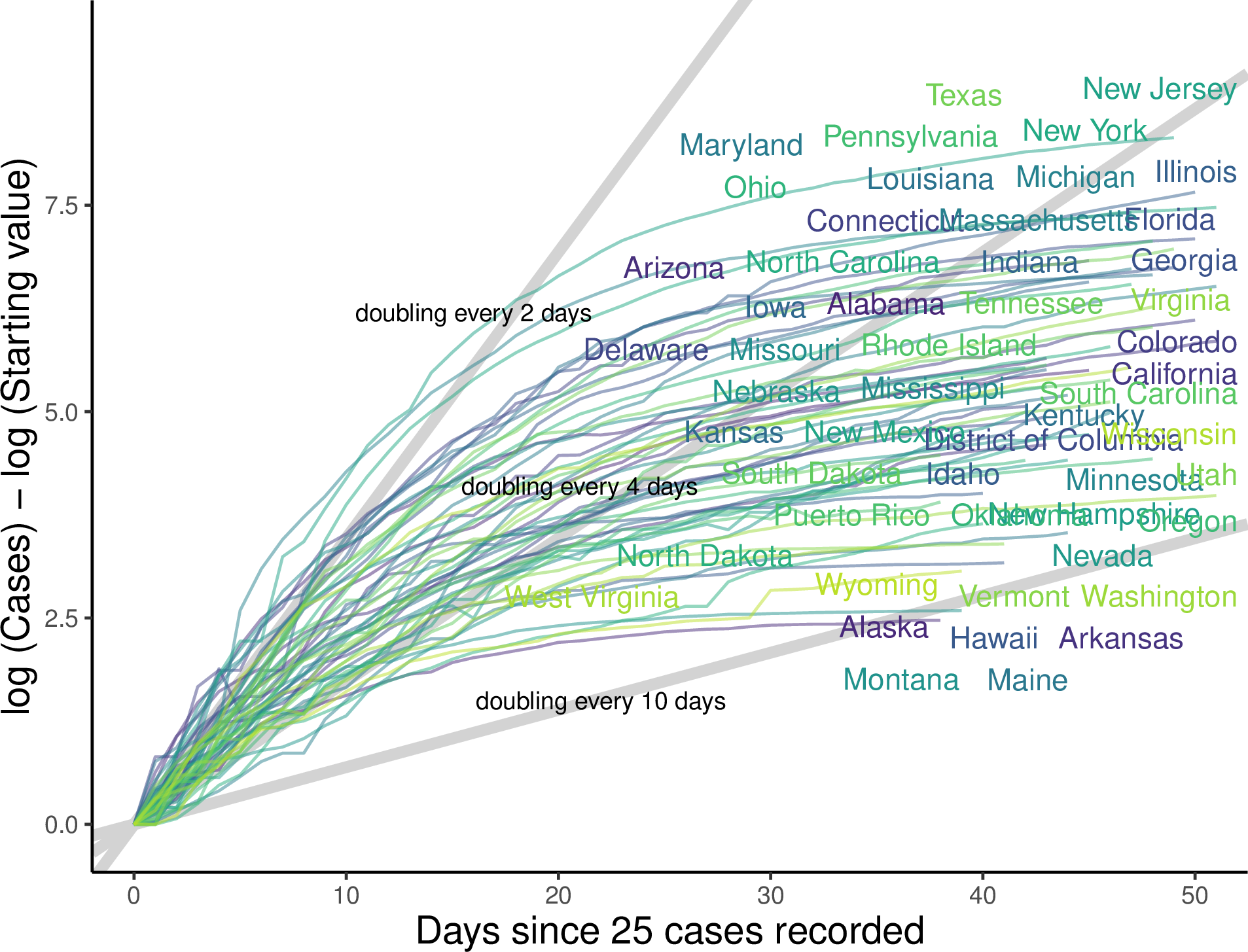

Supplement: S2 Fig — (TIF) [file pone.0240648.s002.tif]

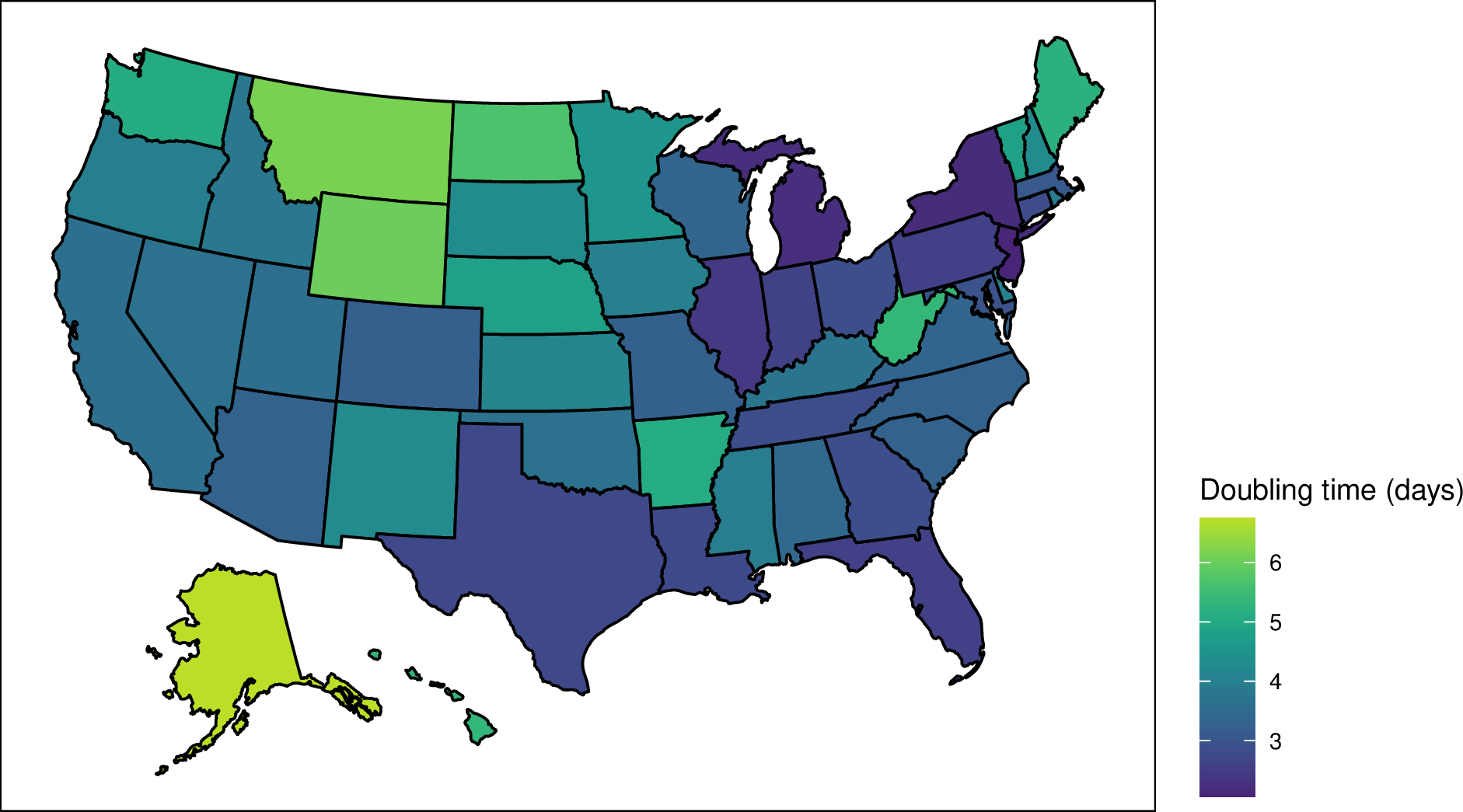

Supplement: S3 Fig — (TIF) [file pone.0240648.s003.tif]

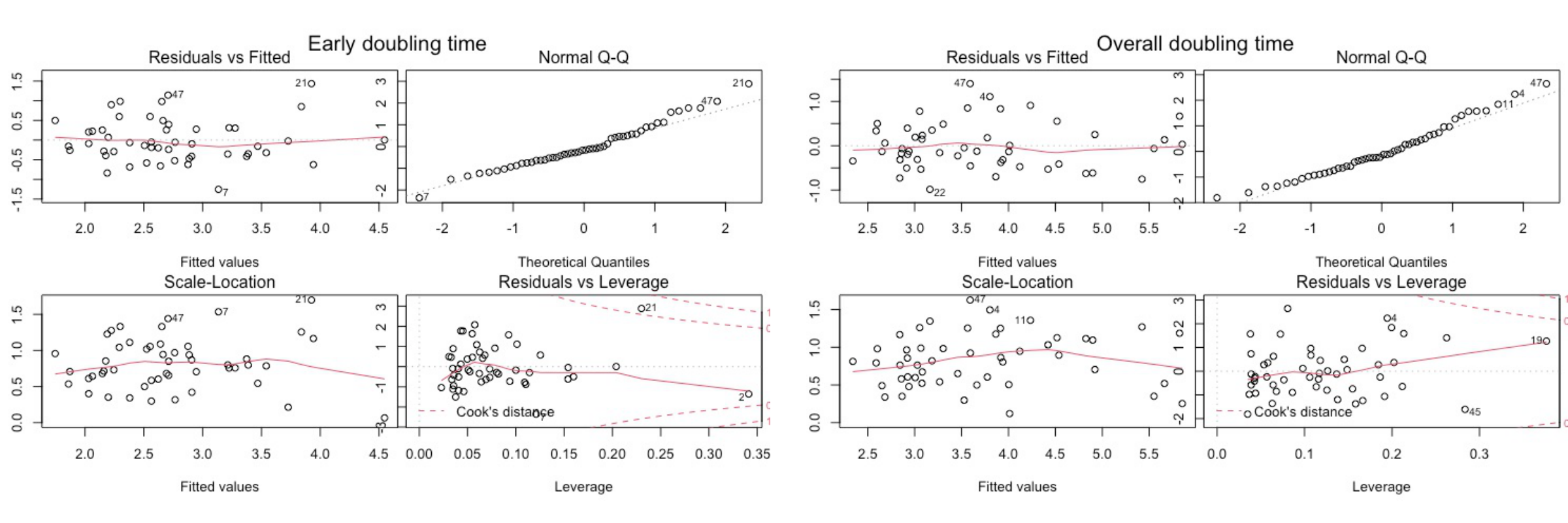

Supplement: S4 Fig — (TIFF) [file pone.0240648.s004.tiff]
