# Supplementary material for: State-level variation of initial COVID-19 dynamics in the United States
Source: PLoS One. 2020 Oct 13;15(10):e0240648. doi: 10.1371/journal.pone.0240648 (PMC7553297; doi:10.1371/journal.pone.0240648)
Supplement: Attachment — Submitted filename: White2020 Response_to_PLoSONE_review.pdf [file pone.0240648.s005.pdf]

Response to Reviewer Comments

The editor agreed with the reviewer that our work was *not technically sound, and our statistical analyses were not performed appropriately and rigorously*. However, the reviewer **does not point out any** methodological flaws, but instead his/her preferences for a specific set of approaches and their own hutches that we must have done something wrong in our models. We can clearly demonstrate that the reviewer's hutches are all in fact incorrect and there are **no methodological flaws** in our work. Our work is both technically sound and the statistical analyses have been performed correctly. We address all these points below and made a couple of changes in the manuscript itself to address any remaining concerns.

Three major points to consider:

- As the reviewer points out, "novelty is not a criterion" and then immediately talks about why this paper is not novel under the guise that the models are "likely misspecified". In reality, we simply use different approaches compared to past work and come to similar conclusions.
- We clearly state that we checked all of our model assumptions using residual plots (which I have included in the attached document). All of the model assumptions of simple linear models are clearly met. This provides strong evidence against the idea that our models are somehow misspecified or inappropriate.
- The reviewer points to other papers with different modeling approaches as evidence that our approaches are not correct. The reviewer provides no evidence as to why our approaches are actually incorrect. We verify other work with a different set of approaches.

Below are specific responses to each reviewer comment. I could provide further detail and analyses for each comment if needed. *Reviewer comments in grey,* and our response in black.

Specific comments:

Reviewer #1: In this paper, the authors explore differences in state-level COVID outcomes and policy responses. There have already been a number of papers exploring exactly the same topic (e.g. Courtemanche et al. 2020) and with much better modeling. I know PLOS ONE has a policy that novelty is not a criterion, but what these other papers highlight is that the models in this paper are very likely misspecified.

I find it very disappointing that a PLoSONE reviewer immediately brought up issues of novelty, especially as the preprint of this paper was published before Courtemanche et al. 2020. The idea that other papers used different models for similar questions in no way suggests the models in our work have been misspecified. We used a different set of approaches to arrive at a similar conclusion as Courtemanche et al. 2020 for the specific result of government interventions and slowing the COVID-19 growth rate.

The authors note the independent variables are highly correlated with one another and only remove them if they are correlated at 0.7 or more. This is still very high collinearity so I'm not sure we can trust the model results. The fact that the authors' note that their results are contrary to those for influenza and for analyses of COVID in other countries, makes me worry that the model is misspecified.

A few different things to unpack here:

A correlation of 0.7 or more is a very common criteria (https://www.ncbi.nlm.nih.gov/pmc/articles/PMC4888898/; https://onlinelibrary.wiley.com/doi/full/10.1111/j.1600-0587.2012.07348.x). Further, even if there were high correlations between variables, this, in and of itself, is not necessarily cause for concern. More importantly, we have to know if we have met model assumptions. As we stated in our manuscript, we were careful to examine residual plots to check all of our model assumptions. **There is nothing of concern in any of the residual plots (see below) for our top fitting models.** This should invalidate concerns about bias. Our models are not misspecified.

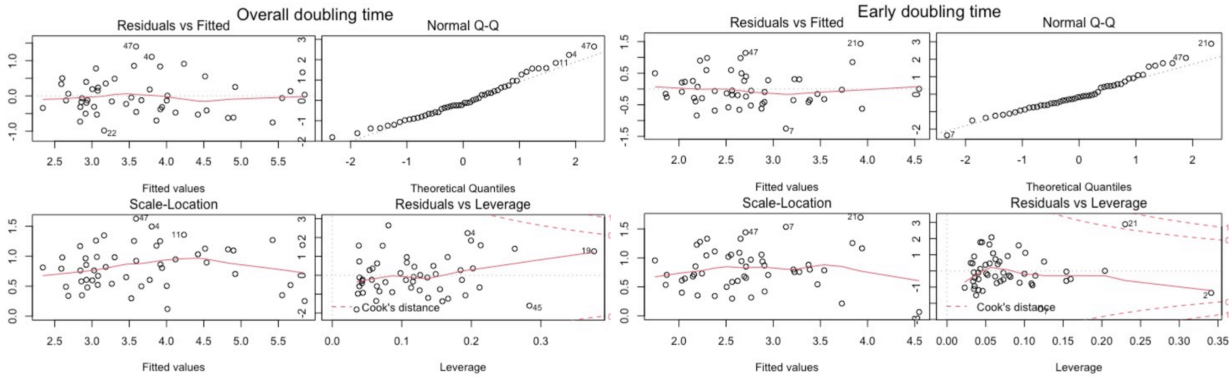

The fact that our results differ from other work is an insulting "piece of evidence" that something must be wrong with our modeling approach. We have one result about population density and COVID-19 that goes against some previous work in China and for influenza. We also cite papers that support our idea in the US specifically.

The policy variable is very crude, equal to 1 if policy interventions happened before 25 cases and 0 if they happened after. Why not use the number of days prior to or before some arbitrary threshold? That's the way the IHME model works (https://covid19.healthdata.org).

Although the policy variables we used were crude as a binary variable, this is actually one of the most intriguing findings of our work. It suggests the timing of intervention may not be that important compared to just the presence of an intervention. We were focused on whether or not an intervention happened early or not as opposed to the specific timing which other papers have focused on.
We don't find comparisons to the IHME model very helpful here. That model is focused on prediction while we are worried about understanding important correlations with the early doubling time. We are asking completely different questions.

I'd also like the authors to justify the arbitrary threshold of 25 cases. And since population varies so much across states, why use number of cases rather than percent of population?

We feel this is the only useful suggestion made by the reviewer. We understand how the threshold of 25 cases could be viewed as arbitrary. We had to choose some starting point and no value will be ideal. Most importantly, our modeling results are consistent only after using a threshold of between 20-25, or greater. For smaller threshold values, the final best fitting model chosen is very sensitive to threshold values. Thus, 25 seemed like a reasonable value.

We felt the 25 cases was an easier value to interpret, whereas cases per percent of population is a bit more abstract. We think the media and other papers focus on the former for this exact reason.

The models very likely suffer from omitted variable bias. A better analysis would forego the state level independent variables and instead use variation over time and fixed effects to account for differences between states.

Every model can suffer from omitting variables. We have no idea why the reviewer feels our particular paper would be "very likely" to suffer from this as they provide no evidence for that idea. We included a specific set of variables based on hypotheses we had about the data. Our goal was not to "p-hack" the data with more and more explanatory variables. The "better analysis" suggested is in no way better but is instead intended for a different analysis and different set of questions. Our goal was to understand how the state-level independent variables were correlated with COVID-19 progression. We use standard statistical tools, with appropriate assumptions met, to do exactly this. **The alternative analysis suggested would not have answered our specific questions.**
