# Supplementary material for: State-level variation of initial COVID-19 dynamics in the United States
Source: PLoS One. 2020 Oct 13;15(10):e0240648. doi: 10.1371/journal.pone.0240648 (PMC7553297; doi:10.1371/journal.pone.0240648)
Supplement: Attachment — Submitted filename: Response To Reviewers.pdf [file pone.0240648.s006.pdf]

Response to Reviewer Comments

Dear Dr. Jeffrey Shaman,

Thank you for giving us the opportunity to re-submit a revised draft of our manuscript titled, "State-level variation of initial COVID-19 dynamics in the United States". We thank the reviewers for their constructive comments on the manuscript. We have included additional text and citations, modified the analyses and figures, and clarified areas of the methods.

Below we detail the changes we made to our manuscript in response to each reviewer comment. We leave the *original reviewer comments in red, italicized font*. Our responses are in black, Roman font. We have also included line numbers of the revised manuscript for each response. In the revised version of the text, we have noted substantive changes in red font.

Journal Requirements:

1) Please ensure that your manuscript meets PLOS ONE's style requirements, including those for file naming.

We have now checked all style requirements.

2) We note that your manuscript is not formatted using one of PLOS ONE's accepted file types. Please reattach your manuscript as one of the following file types: .doc, .docx, .rtf, or .tex (accompanied by a .pdf). If your submission was prepared in LaTex, please submit your manuscript file in PDF format and attach your .tex file as "other."

We prepared our manuscript in LaTex using the PLOS ONE template. All appropriate .tex files are now attached.

3) We note that Figure S3 in your submission contains map images which may be copyrighted.

The map images are not copyrighted. We made them ourselves.

Reviewer 2:

While there seem to be interesting and overall likely well done analyses, there are places where the methods are underspecified to the point where it is unclear if the methods are adequate.

Thanks for the detailed feedback. We address each of the comments more below to address methods concerns. We also include all the code and data required to rerun our analyses.

Major points

While I understand the need for stepwise regression and concerns about correlation. It is unclear how the authors selected variables when there were highly correlated variables. If two variables are highly correlated, it would be likely (although not absolutely true) that they would either both meet stepwise inclusion or neither

We have now added text on line 114 to clarify this point. The only highly correlated variables were the demographic variables. For example, percent of population in rural areas was very strongly correlated with percent of population in urban areas. We ended up just choosing one variable if there were several correlated. There was no strong reason behind each choice. However, the variables that were strongly correlated had similar interpretations, as in the example above about population percentage.

Please clearly list all candidate predictors (state variables as well as policies) that were considered as part of the stepwise regression

These are now listed in the Table 1 caption, and the list starting on line 120.

Please provide more context for the inclusion of the volunteer rate in the methods. This inclusion seems interesting but the reader doesn't have context for this variable. Similarly, the tightness score (this comes through a little more in the discussion, but explaining in methods would be helpful)

We have now added additional context on lines 63-70 and 132. In short, we hypothesized that volunteerism could be a measure of how willing someone would be to accept restrictive policies that might be beneficial to the community overall.

Please give more information regarding the gathering limitations. You say "usually to 10 people" but the reality is that almost all states started at 250 or 100, then decreased to 50, then 10, some went to smaller numbers too. So what date are you using? The first date or the date the states went to 10 people?

We address this point on line 150: "We follow Adolph et al. and use the first date of gathering restrictions announced, regardless of the size of the gatherings restricted."

The references (and at times the text) need to updated. I believe publishing on the beginning of the pandemic is useful but putting these findings into context of other publications around the same time period is useful. A prior reviewer mentioned the Courtemanche manuscript, there is also the Hsiang manuscript in Nature. This submitted manuscript confirms many of the findings of Auger re schools in JAMA. Thus, I think the concerns raised by prior reviewers that the model is not correctly specified are not founded.

We have now added several references and updated text (see lines 21, 70-78, 213) to address this.

References

B. M. Althouse, B. Wallace, B. Case, S. V. Scarpino, A. M. Berdahl, E. R. White, and L. Hebert-Dufresne. The unintended consequences of inconsistent pandemic control policies. *arXiv preprint arXiv:2008.09629*, 2020.

K. A. Auger, S. S. Shah, T. Richardson, D. Hartley, M. Hall, A. Warniment, K. Timmons, D. Bosse, S. A. Ferris, P. W. Brady, et al. Association between statewide school closure and covid-19 incidence and mortality in the us. *JAMA*, 2020.

Z. Du, X. Xu, L. Wang, S. J. Fox, B. J. Cowling, A. P. Galvani, and L. A. Meyers. Effects of Proactive Social Distancing on COVID-19 Outbreaks in 58 Cities, China. *Emerging Infectious Diseases*, 26(9):2267–2269, Sept. 2020. ISSN 1080-6040, 1080-6059. doi: 10.3201/eid2609.201932.

L. C. Lopes-Júnior, E. Bomfim, D. S. C. da Silveira, R. M. Pessanha, S. I. P. C. Schuab, and R. A. G. Lima. Effectiveness of mass testing for control of covid-19: a systematic review protocol. *BMJ open*, 10(8):e040413, 2020.

N. W. Ruktanonchai, J. Floyd, S. Lai, C. W. Ruktanonchai, A. Sadilek, P. Rente-Lourenco, X. Ben, A. Carioli, J. Gwinn, J. Steele, et al. Assessing the impact of coordinated covid-19 exit strategies across europe. *Science*, 369(6510):1465–1470, 2020.

J. Solis, C. Franco-Paredes, A. F. Henao-Martínez, M. Krsak, and S. M. Zimmer. Structural vulnerability in the us revealed in three waves of covid-19. *The American journal of tropical medicine and hygiene*, 103(1):25, 2020.

T. K. Tsang, P. Wu, Y. Lin, E. H. Lau, G. M. Leung, and B. J. Cowling. Effect of changing case definitions for covid-19 on the epidemic curve and transmission parameters in mainland china: a modelling study. *The Lancet Public Health*, 2020.

B. Xu, B. Gutierrez, S. Mekaru, K. Sewalk, L. Goodwin, A. Loskill, E. L. Cohn, Y. Hswen, S. C. Hill, M. M. Cobo, et al. Epidemiological data from the covid-19 outbreak, real-time case information. *Scientific Data*, 7(1):1–6, 2020.

In terms of updating the manuscript, the second sentence discusses the case counts as of April 29th. The reader will be confused by this statement given how different we are currently. I see why you are presenting this data as this is at the end of the study period but you need to first introduce the study period and then give context of the numbers.

This has been corrected on line 21.

Similarly, you need to at least acknowledge the latest phase of the pandemic (lines 40-45) there is another rise in cases/deaths after reopening. I understand that you are not focused on that time period but it is strange to not acknowledge it. In fact you state the numbers have "slowed since mid-March" (line 50) which is simply not true.

We certainly agree. Some of the numbers and text were out of date as we originally submitted this manuscript back in May. We have now corrected this on 81.

Results—Table line 123. You state that demography, education, etc are poor predictors and cite table 1; yet none of these variables are in table 1.

This is an important point that we now clarify in the table caption. These variables were such poor predictors that they were not selected by the final models after a model selection process.

Please put in context the parameter estimates from Table 1 into the text. What does a doubling time parameter estimate of 0.492 for schools mean??? This would be helpful for the entire table.

We have now included this context in examples on lines 202 and 215.

It is kind of a big deal that testing wasn't accounted and is only listed as a limitation. The statement "overall doubling time was not strongly correlated with overall tests rates for each state" line 135 is woefully underspecified. (the text says figure 4, but It appears to refer to figure 5). The reader has no idea what is meant by test kits per capita. Is this the number of tests per people over some time? What time period? Why not just include the daily testing rate as a time varying covariate?

We now include testing rates as a covariate in our models (Table 1). It was never chosen in the best models after model selection. We have now modified the figure to just read testing rate, which is the total number of tests conducted at 7 days or three weeks, depending on the model. We modified the text on lines 182 and 246 to address this.

Figures 3 and 4 would be improved with some sort of correlation coefficient in each panel. It is unclear if all of the panels in figure 4 were considered as candidate covariates. Why aren't business restriction, stay at home mandates, and number of state actions in the final model? Were they not retained in stepwise? Were they too highly correlated?

We have now included a correlation coefficient on each of these figures. We have also now only plotted the covariates that were included in the models described in table 1. The reviewer is correct that several variables were not selected in the best model for the stepwise model selection process. We expand on this in the caption of table 1.

Specific points

Abstract *The abstract states "we examine how commonly used epidemiological metrics differ for each individual state within the US during the initial COVID-19 outbreak." Yet, the manuscript does not report out individual state level results so this sentence should be revised.*

We are not sure what the reviewer meant with this comment. All of our analyses highlights the individual state level differences in doubling time and other variables. It is true that we did not look at the dynamics within each state.

Methods *"The early doubling time should not be severely affected by government interventions s these were rarely implemented that early in the course of the epidemic" (line 65-66) I think the sentiment of this statement is true... but it is likely more that the effects of the policies wouldn't take place that fast. Several states closed schools and implemented gathering bans when they had very very few cases (e.g. West Virginia closed schools with 0 cases). Given that it would take 10+ days for policy change to impact coronavirus spread (see lag in model derived by Auger, JAMA), I believe this is a reasonable approach; however, you may want to tweak the language because it isn't that the policies weren't implemented it is more they wouldn't have had time to take effect.*

We modified this sentence on line 102.

Results *"We found that doubling times for all states increased with time and that heterogeneity between states was reduced" line 109-110. This seems very subjective. It is strange not to quantify this in some way. How much did they change?*

On line 155, we clarify this comparison using Figures 2a and 2b.

Discussion *The first paragraph of the discussion is a bit long and feels tangential to the main point of the manuscript. Perhaps this could be largely reduced and additional references regarding emerging COVID studies (listed above) could be incorporated. There are some nice additions (or considerations) in this manuscript which haven't been in other models (tightness, volunteer rate, etc). So highlighting these is appropriate (as you do in lines 170-180).*

Starting on line 185 we have simplified the text.

Supplemental figure 3 *This one is a bit confusing to me. You talk about the changes in the doubling time throughout the study period; yet, this is just a single number per state... so when is this from? Early? The average of the study period?*

In the figure caption, we now clarify that this map was the doubling time for the first three weeks since 25 cases in each state.

Reviewer 1:

The reviewed paper presents an interesting analysis on how state-level variation can explain the early pandemic dynamics of COVID-19. The analysis appears to be logical and statistically sound. I don't have any major comments, but have a few minor comments that I think could enhance the manuscript.

Thank you for the feedback.

Minor comments

The authors note: "We show that early non-pharmaceutical government actions were the most important determinant of epidemic dynamics." I think this statement can be interpreted in two ways: (1) government actions need to happen early to impact the epidemic dynamics, or (2) the government actions that happened early had the largest impact on subsequent epidemic dynamics. These have two different interpretations in my mind, that the specific timing in the early phase matters or that implementing them early at all matters. I believe the authors mean the latter, but I think it would be useful to clarify, maybe something like this?: "We show that non-pharmaceutical government actions during the early phase of the epidemic were the most important determinant of epidemic dynamics." One could reference previous research from China that agrees with this conclusion as well (I know it likely wasn't published when this was written): `https://wwwnc.cdc.gov/eid/article/26/9/20-1932_article`

On line 9, we have made this change with the suggested text and added the citation to the discussion.

Figure 5: The authors note: "The overall doubling time was not strongly correlated with overall tests rates for each state (Fig. 4)." Did the authors compare with testing rates as a raw number rather than per capita? I wonder if there would be a stronger correlation, as the early pandemic dynamics with small numbers might not have any per capita impact.

We now include testing rates as a covariate in our models (Table 1). It was never chosen in the best models after model selection. We also examined the raw number instead of per capita and this was less strongly correlated.

In general, I found it unintuitive to follow the doubling time results. E.g. on line 120: "We found that population density, flu vaccination rates, and wealth were all positively correlated with doubling times" I would suggest rephrasing for clarity for these types of results throughout to something like: "Comparing the doubling times across states, we found that increasing population density, flu vaccination rates, and wealth were all associated with slower epidemics" – I'll leave it to the authors to decide if they agree

We have now corrected this on line 163.

Line 120 indicates that all three variables are positively correlated with doubling times, but the results in Table 1 have different signs for population density from the other two variables, so I think there might be a typo?

Same as above.

Line 173, I'd add that tightness was negatively correlated with doubling time.

This has been corrected on line 181.

Typo in line 180: "We hypothesis this may be the result of people in tight cultures finding it more difficult to adjust their behavior when new rules are imposed."

This has been corrected on line 230.
